# Is the Cloze Procedure Appropriate to Evaluate Health Literacy in Older Individuals? Age Effects in the Test of Functional Health Literacy in Adults

**DOI:** 10.1155/2014/194635

**Published:** 2014-09-11

**Authors:** Raymond L. Ownby, Amarilis Acevedo, Drenna Waldrop-Valverde, Robin J. Jacobs

**Affiliations:** ^1^Department of Psychiatry and Behavioral Medicine, Nova Southeastern University, Fort Lauderdale, FL 33314, USA; ^2^Center for Psychological Studies, Nova Southeastern University, Fort Lauderdale, FL 33314, USA; ^3^Nell Hodgson Woodruff School of Nursing, Emory University, Atlanta, GA 30322, USA

## Abstract

Health literacy has received increasing attention because of its importance for older individuals' health, as studies have shown a close relation between older individuals' health literacy and their health. Research also suggests that older individuals have low levels of health literacy, but this finding is variable and may depend on which health literacy test is used. Older individuals assessed with the Test of Functional Health Literacy (TOFHLA) score lower than younger individuals, but a previous study suggested that this may result from age-related differential item functioning (DIF) on the TOFHLA. The study reported here assessed age-related DIF in a sample of community-dwelling volunteers. Twenty-two percent of items were differentially more difficult for older individuals independent of their overall ability, and when these items were eliminated from the total score, age differences were no longer found. Performance on a working memory task predicted older but not younger individuals' performance on the age-related items. At least part of older individuals' apparent deficits in health literacy when assessed by the TOFHLA may be related to DIF on its items. The TOFHLA, and any measure that employs the cloze procedure to evaluate reading comprehension, should be used cautiously in older individuals.

## 1. Introduction

Over the past several decades, research on health literacy has become increasingly relevant to gerontologists, as studies have shown that older adults' health literacy is an important factor in their health status and service utilization [1–4]. Health literacy has been defined as “*… the degree to which individuals have the capacity to obtain, process, and understand basic health information and services needed to make appropriate health decisions*” [5] and is related to older individuals' ability to use health information for decision making. Studies have shown that level of health literacy is related to use of health services and measures of disease control [1, 6]. It has also been related to increased risk for hospitalization in Medicare beneficiaries [7] and even to greater risk of death [8, 9].

Several studies have found that individuals 65 years of age and older perform at lower levels on measures of health literacy compared to younger individuals [10, 11]. The National Assessment of Adult Literacy (NAAL) was a population-based study of literacy that included a health literacy scale [10]. Age comparisons showed that those 65 years of age and older performed at lower levels compared to participants who were younger. Studies with other measures, including the widely-used Test of Functional Health Literacy in Adults or TOFHLA [12], have also found age-related decrements in performance [13]. On the other hand, some studies with another widely-used measure of health literacy, the Rapid Estimate of Adult Literacy in Medicine, or REALM [14], have not found age-related differences [9, 15–17]. Given the link between health literacy and health, a better understanding of reasons underlying older individuals' poorer performance on some but not all measures of health literacy might yield information that could inform the development of interventions to improve it.

One possible source of age-related differences in performance on tests of health literacy is the type of response required by the measure. The TOFHLA, for example, uses the cloze procedure to evaluate comprehension of health-related written material [18, 19]. With the cloze procedure, the person evaluated is asked to read a text passage and supply words that have been deleted and replaced with a blank (e.g., “The sky is —”). In the case of the TOFHLA the authors deleted every 5th to 7th word from three passages which progressively increased in level of difficulty [12]. Although the cloze procedure was created to assess text readability [19], it has been widely used as a device for measuring comprehension [20] and is incorporated in other health-related measures [21], although it has been criticized as inappropriate for use with low literacy individuals [22, 23] and for routine use in healthcare settings [24].

The cloze format may be difficult for older adults due to its demands on cognitive abilities that decline with age, including verbal fluency and working memory [25]. Studies have shown that cloze performance may depend on working memory and that it modifies the relation between age and general cognitive ability in cognitive task performance [18, 26]. Other authors have also demonstrated that working memory may differentially affect reading comprehension in older individuals [27, 28], still further implicating it as a factor in performance on tests that use the cloze procedure. Finally, a previous study showed age-related differential item functioning (DIF) on several TOFHLA items in individuals over 50 years of age who were treated for HIV infection [29], suggesting that some TOFHLA items may be harder for older individuals than younger individuals even when they have the same overall level of health literacy. This study's small sample size and the existence of disease-related cognitive deficits in study participants, however, make it difficult to generalize its results to other groups.

Using the cloze procedure to assess comprehension may thus inadvertently create a test with items that are differentially more difficult for elders, confounding the assessment of health literacy with age-related changes in cognition. Apparent age-group differences may result that suggest that elders' health literacy skills are lower than they actually are. This possibility can be tested by assessing whether test items display differential item functioning or DIF related to age [30]. A test item is said to exhibit DIF when it is more difficult for a member of one group than another (e.g., men versus women, blacks versus whites, or older versus younger) even though each person's underlying overall ability is the same. If age-related DIF is present in the items of the TOFHLA, some items would be more difficult for older than younger individuals even though they have the same overall ability. This could occur because the DIF-related items make demands on a cognitive ability such as working memory that declines with increasing age. In essence, items with age-related DIF would thus evaluate two abilities in older individuals (health literacy and working memory) while ostensibly assessing only one (health literacy).

The purpose of these analyses was therefore to evaluate whether items on the TOFHLA show age-related DIF using a nonparametric approach to identify DIF due to our sample size. We also chose to explore the possibility that working memory would be related to performance on items showing DIF using standard parametric regression analysis and test whether taking age-related DIF into account would explain age-group differences in performance on the TOFHLA by comparing the performance on age-based groups on the measure with and without items that show age-related DIF.

## 2. Method

### 2.1. Overview

In this study we drew on data collected in a larger study whose purpose was to develop and validate a new measure of health literacy [31, 32]. Participants whose ages ranged from 18 to 86 provided demographic information and completed the TOFHLA and a battery of cognitive and academic measures. These data thus provided the basis for an assessment of differences in TOFHLA performance between those 65 years of age and older compared to those who were younger while taking into account potential confounders that included education, general verbal ability, and basic reading skills. Age-related DIF was first assessed in the items of the TOFHLA as described below. As we hypothesized that working memory might be a factor in age-related DIF, the relation of performance on a measure of working memory (digit span backward) to performance on the DIF-related items was evaluated in younger and older individuals. Finally, to evaluate the relevance of DIF to age group differences on total TOFHLA reading scores, differences between groups were tested on the sum of all TOFHLA comprehension items and a recalculated total score with DIF-related items was eliminated.

### 2.2. Participants

**Figure 1:**
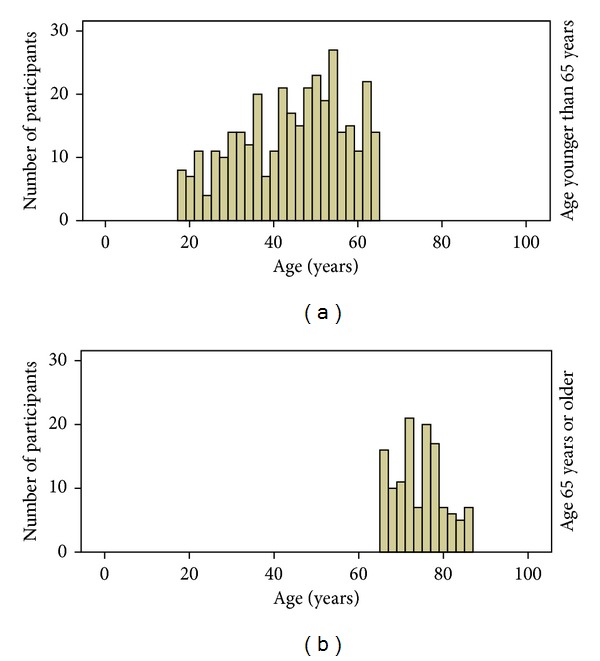
Age distribution of participants by group.

Participants were community-dwelling volunteers aged 18 and older recruited through flyers in community centers, word of mouth, and from previous studies. As the purpose of the parent study was to develop a new measure of health literacy and to provide preliminary norms for it, participants were selected to represent a wide range of ages and educational levels and to be distributed across gender and race [31, 32]. Of the 167 participants who were younger than 65, 70 were white and 97 were black and 75 were men and 92 were women. Of the 69 participants who were 65 years of age or older, 54 were white and 15 were black and 19 were men and 50 were women. It can be seen that the average age of the younger group was approximately 43 while that of the older group was approximately 74 years.  Figure 1 shows the number of individuals at each age in the two age groups and additional descriptive information is provided in Table 1.

### 2.3. Measures

As part of a battery of ability and skill measures, the complete TOFHLA, including 50 reading comprehension items, was administered. The reading comprehension portion of the TOFHLA requires the person assessed to read three paragraphs of health-related material whose difficulties progressively increase. The first paragraph is based on simple instructions on how to prepare for an X-ray, the second focuses on patient rights and responsibilities in a government-supported health insurance program, and the third is drawn from an informed consent form for a surgical procedure. Every fifth to seventh word is removed from the text and indicated by a line; under the line, several options, one of which is correct, are presented. Participants were tested according to the standard directions for the measure [12] and were allowed 20 minutes to complete the reading comprehension questions. Their responses were categorized as right or wrong according to the test's administration instructions [12].

General verbal ability was assessed using the Verbal Composite score of the Woodcock-Johnson Psycho-Educational Battery [33]. This measure evaluates word knowledge, general information, and verbal reasoning in a series of tasks that yields a single score. Basic reading skills were assessed with the Passage Comprehension subtest of the Woodcock-Johnson Battery. This subtest uses items based on one or two sentences to evaluate a person's ability to understand what is read. Working memory was assessed with the Digit Span Backward subtest of the Wechsler Adult Intelligence Scale, 3rd edition [34].

Participants completed individually-administered cognitive measures in a single session while completing a group of self-report measures in a second session. Sessions were randomized to minimize order effects (i.e., some participants completed self-report measures first and then cognitive measures, while other completed cognitive measures first). The order of test administration within sessions was fixed. The Digit Span test was administered first in the battery because of its simplicity; it was judged as a good warm-up task compared to later measures that included the TOFHLA.

### 2.4. Analyses

#### 2.4.1. Assessment of DIF

**Figure 2:**
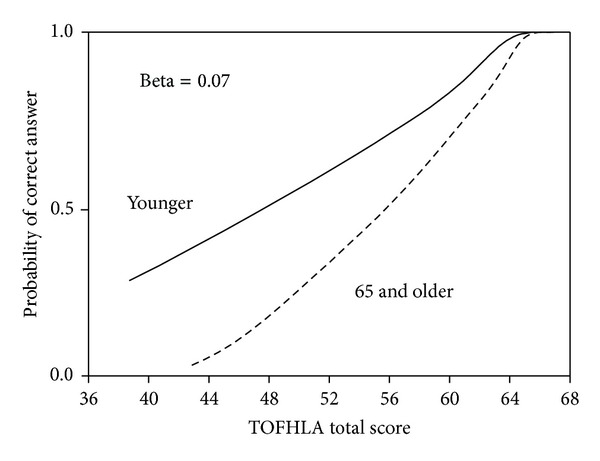
Item response curve for TOFHLA item 41 illustrating DIF between younger and older individuals.

Standard approaches to evaluate DIF typically employ tests of group-based differences based on large-sample parametric statistics [35]. Some experts suggest that the sample size for this type of analysis should be at least 1,000 [30] and a simulation study showed that DIF detection rates may be suboptimal even with a sample of 500 when comparison groups are unequal in size [36]. As the available sample was substantially smaller than required for these techniques, an alternate approach to item assessment was followed using nonparametric item response theory (IRT) techniques. Age-related DIF was assessed with the beta index provided in TestGraf, a free nonparametric IRT program [37]. TestGraf produces item characteristic curves which provide a graphic representation of the probability of individuals in different groups obtaining a correct score on an item in relation to their overall ability. If DIF is not present, the curves for each group should be congruent or nearly so, but if DIF is present, the two curves will diverge (see Figure 2). The beta index is a numeric representation of the area between item response curves for two groups. Cutoff scores for the beta index to identify DIF for small sample sizes have been provided in a simulation study by Zumbo and Witarsa [38], who also showed that standard approaches such as the Mantel-Haenszel chi-square test have inadequate power to reliably detect DIF in small samples. The free software package jMetrik (http://www.itemanalysis.com/) was used to calculate item difficulties, standard deviations, and discriminations (defined as the correlation of each item with the total scale score).

The sample was divided into two groups based on age less than or greater than or equal to 65 years. Age 65 was used for comparison as it has most often been used in research on age-related cognitive abilities and is linked to both age-related changes in working memory [39] and reports of older individuals' lower levels of health literacy [3, 13]. Items that exceeded the critical value of beta for the available sample size [38], thus indicating at a probability of less than 0.05 that DIF was present, were tabulated.

Although we used a nonparametric strategy to evaluate age-related DIF, our sample size was sufficient to allow us to assess the overall effect of DIF on group scores with a standard parametric strategy, analysis of covariance (ANCOVA). This strategy enabled us to correct for between-group differences in important covariates (e.g., education and reading skill) while comparing group mean performances. Each group's scores for all TOFHLA reading items and the corrected total with DIF-related items removed were calculated and compared in analyses that controlled for general verbal ability and reading skills. The possibility that observed age group based differences resulted from older participants' inability to complete all items of the TOFHLA was evaluated using chi-square analyses; we followed up this assessment with a similar analysis of the relation educational achievement less than high school level and failure to complete the TOFHLA. As it was hypothesized that working memory would account for group differences on items, its effect on between-group differences was also evaluated using another parametric strategy by assessing its relation to performance on the DIF-related items in both age groups in regression analyses. As the focus of these analyses was on the possibility that items using the cloze procedure were differentially more difficult for individuals aged 65 and older, TOFHLA Numeracy items were not included as they do not utilize the cloze procedure.

All study procedures were completed under a protocol approved by the Nova Southeastern University Institutional Review Board.

## 3. Results

Descriptive statistics for participants are presented in Table 1. It should also be noted that each of group's scores on the TOFHLA Reading is similar, in spite of previous reports of significantly lower performance among older individuals. This fact can be attributed to the overall higher levels of education and basic reading skill in the older group, an issue that we addressed in group comparisons (reported below) by taking these factors into account in analysis of covariance (ANCOVA) models.

Item difficulties (percent answering correctly), discriminations (item-total correlations), and DIF analyses (beta values representing the area between item response curves for age groups) are presented in Table 2. Items with beta values greater than the *P* < 0.05 cut point of 0.0421 are in bold font [38]. Eleven of the 50 items (22% of the total) showed evidence of age-related DIF. An example of a nonparametric item characteristic curve for an item with DIF (item 41 in TOFHLA paragraph C) is presented in Figure 2. The figure includes item curves for younger and older individuals; each curve shows the probability of a participant answering the item correctly (left axis, ranging from 0 to 1) in relation to his or her overall ability. As Figure 2 shows, older individuals have a lower probability of answering this item correctly even when their total score was the same as a younger person's total score. Older individuals would thus have lower overall scores on the TOFHLA as a result of age-related DIF on these 11 items.

Table 3 presents regression models assessing factors related to performance on the sum of the DIF-related items for younger and older participants. For younger individuals, general verbal ability, reading skills, and years of education were related to performance on the DIF-related items (model 1). The addition of working memory to the model (model 2) did not significantly improve its relation to performance on the items (change in *R* ^2^ = 0.01, *F* (1,149) = 1.86, *P* = 0.18). For individuals 65 or older, reading skills and education, but not general verbal ability, were related to performance on the DIF items. The addition of working memory (model 2) resulted in a significant improvement in the model's relation to performance on the DIF items (change in *R* ^2^ = 0.04, *F* (1,57) = 5.82, *P* = 0.02). These results suggest that while working memory may not have been an important determinant of performance on the DIF-related items for younger individuals, it was significant for older participants.

We evaluated the possibility that between-group differences on the TOFHLA resulted from older individuals not completing the test, as the standard directions for the TOFHLA reading section specify a time limit for its completion. Nineteen participants did not finish the TOFHLA because of the time limit, 14 in the younger and 5 in the older groups. We assessed the relation of not completing the TOFHLA to age group and level of education (having completed high school compared to less than high school education) via chi-square analyses. The relation between age group and not completing the TOFHLA was not statistically significant (*χ* ^2^  (df = 1) = 0.09, *P* = 0.77), but the relation between less than high school educational attainment was (*χ* ^2^  (df = 1) = 4.46, *P* = 0.04).

Finally, the impact of DIF on overall performance on the TOFHLA reading scale was evaluated by testing between-group differences on the TOFHLA scores with and without the DIF items. Differences were evaluated in ANCOVA models that included general verbal ability, reading skill, and education as covariates. There was a significant difference between older and younger participants for the score on all 50 reading items (mean score for younger = 46.5; mean for older 43.8 for a mean difference of 2.4 points; *F* (1,231) = 17.26, *P* < 0.001). This difference was no longer significant after removal of the 11 items with DIF (mean score for younger = 39.6; mean for older 39.0 for a mean difference of 0.6 points; *F* (1,206) = 2.62, *P* = 0.11). These results thus suggest that a portion of age-related differences on the TOFHLA reading scale resulted from the older group's performance on DIF-related items.

## 4. Discussion

These results confirm a previous report of age-related DIF in a potentially clinically relevant number of items of the TOFHLA reading scale [29] and extend this finding by investigating the extent to which working memory may be a factor in age-related DIF and testing its impact on age-related differences in TOFHLA scores. More than 20% of TOFHLA reading items showed age-related DIF; seven of these 11 items also were found to display age-related DIF in a previous study with HIV-infected patients younger or older than 50 years of age [29]. In the present study, working memory as measured by digit span backwards was significantly related to older participants' performance on these items not to that of younger participants. Further, when the 11 DIF-related items were removed from the total TOFHLA reading score, a significant age group difference was no longer present. Since working memory declines with age, an implication is that at least a portion of reported age differences in health literacy, at least for studies that used the TOFHLA, may be the result of its reliance on the cloze procedure that is related to working memory [18] rather than health literacy itself.

The finding of age differences on some health literacy measures while not on others also raises an important issue about how health literacy should be operationally defined. Ownby et al. [40] suggest that health literacy should be defined as a combination of basic cognitive abilities, reading skills, and health knowledge. Within this framework, our findings can be interpreted as showing that working memory may be a more important aspect of health literacy for older than younger individuals. To the extent that working memory is integral to health literacy, individuals 65 and older can be said to have lower levels of health literacy. When health literacy is defined as the ability to read health-related words aloud, however, (as in the REALM) it appears likely that older individuals may not be at a disadvantage. Another study [41] also showed that specific cognitive abilities may have a differential impact on S-TOFHLA performance, similar to the finding reported here. This finding may also reflect the fact that sight word recognition skills such as those assessed in the REALM are often considered resistant to age-related change [42, 43]. The definition of health literacy most commonly used inherently assumes that a patient has necessary basic cognitive abilities and academic skills; these findings emphasize the importance of explicitly recognizing their impact on performance on health literacy measures. A further study is needed to determine which approach to defining and measuring health literacy has the greatest usefulness.

Limitations of these analyses include the relatively small sample size used to assess DIF in the TOFHLA. As discussed above, traditional approaches to assessing DIF may require much larger samples and the approach used in these analyses (nonparametric IRT) is less well established. The selection of working memory as a possible mechanism for observed age-related DIF, while supported by previous work on the cloze procedure [18] and cognitive aging [25], neglects the possibility that some other factors not included in analyses may have been responsible for observed differences in item functioning among older individuals. Still another possibility is that older participants might have become more easily fatigued during assessment activities, thus affecting their responses on the TOFHLA. Since our finding of DIF was not consistent across all items of the TOFHLA, however, this possibility appears unlikely. Our participants completed the study in two sessions, randomized to reduce the possibility of order effects. Since order of administration of the test battery was randomized between mornings and afternoons, we believe that fatigue is an unlikely explanation for the finding of DIF, although it must be acknowledged that the majority of the items that show DIF are in the last third of the test. The finding of age-related DIF on the TOFHLA reading items raises the question as well of alternative explanations for the finding. It is possible, for example, that the content of sections B and C of the TOFHLA Reading scale is less familiar to older compared to younger individuals. We thus acknowledge that other explanations, either instead of or in addition, to age-related cognition might account for these findings.

Another important issue that is raised by these findings is the question of why older individuals perform more poorly on other health literacy tests that do not use the cloze procedure. A study discussed above [17] with the Newest Vital Sign, or NVS [44], found age differences in performance on it but not on the REALM. In the NVS, individuals' health literacy is assessed through their ability to respond to a series of questions about a nutrition label. Perhaps the largest study of health literacy ever done, the National Assessment of Adult Literacy, included items assessing health literacy and found that it was significantly lower in participants 65 and older [10]. One possible explanation for these findings also involves the response format of these measures, all of which require verbal fluency and executive functions in addition to reading and health knowledge for responding correctly. In contrast to the REALM, the NVS and NAAL surveys require the person assessed to produce a new verbal response to questions (compared to the REALM which only requires reading words aloud, a skill that is often highly practiced among older individuals). Since verbal fluency and executive functions, like working memory, may decline with age [45], it can be speculated that findings of age-related decrements on these measures may also reflect at least in part their response formats. In this connection it is also worth noting that others have reported that executive functions are related to performance on the S-TOFHLA [46, 47]. It is thus possible that age-related cognitive changes in addition to working memory may have an impact on older individuals' performance on these measures. It will thus be important to further evaluate the cognitive abilities related to health literacy in individuals across age groups in order to better understand what health literacy tests actually measure. Investigations in this area might also inform efforts to create age-appropriate health education materials for older individuals. For example, cognitive load theory [48, 49] would dictate that taking older individuals' level of working memory into account would be important in the design of instructional materials.

### 4.1. Practice Implications

These finding have implications for the assessment of health literacy not only in English but also in other languages. The cloze procedure, for example, has been used to assess reading comprehension in languages other than English, such as Japanese [50], and there is considerable interest in measuring health literacy in countries outside North America, for example, Sorensen et al. [51]. These findings may thus be relevant for the assessment of health literacy in languages other than English.

An additional practice implication is that it may be more appropriate to use measures less sensitive to cognitive aging when evaluating health literacy in individuals 65 and older, especially as other analyses have shown that performance on the TOFHLA is closely related to cognitive abilities that show age-related cognitive decline [40], such as fluid cognitive abilities like nonverbal reasoning [52]. In this study, the REALM was shown to be less dependent on general cognitive abilities and more closely related to reading and health knowledge. This suggests that the REALM may be a better tool than the TOFHLA for assessing the skills needed to understand health-related material. A new measure of health literacy, FLIGHT/VIDAS (developed as part of the study reported here), was created to explicitly exclude items that showed age-related DIF during initial development and testing [31].

Several other approaches are available that allow for an approximation of older individuals' health literacy while not requiring the same cognitive skills as does the TOFHLA. The Brief Health Literacy Screen [53] allows an assessment of a person's health literacy skills by self-report; the individual to be assessed is asked about the degree of difficulty he or she experiences with several forms of written health information. While this approach has the advantage of providing an estimate of an individual's subjective perception of his or her health literacy, it is not a direct assessment of an individual's performance with health-related materials and does not take into account factors beyond health literacy that might affect a patient's self-report. It should be noted that the correlation between standard measures of health literacy and the Brief Health Literacy Screen questions is substantially smaller than that between direct assessments of health literacy [31, 54]. The Demographic Assessment for Health Literacy [55] provides an estimate of a person's score on the short version of the TOFHLA [56] based on his or her demographic characteristics. This measure's regression-based approach may be useful in large group analyses (as was intended by its developers) but it is not a direct individual assessment of a person's performance.

It may be reasonable to conclude, as have others, that it is important to consider cognitive task demands and purpose when selecting a health literacy measure [54]. These results thus further support others' observations of the variable relations of common measures of health literacy with age and the importance of specific cognitive abilities for performance on measures of health literacy [41]. Given the evidence of age-related DIF on a substantial number of items in the reading comprehension subtest of the TOFHLA, it may be incorrect to interpret findings from it alone as proof that older individuals have lower levels of health literacy. The TOFHLA and any measure using the cloze procedure to assess comprehension should be used cautiously in older individuals; the REALM or FLIGHT/VIDAS may be more appropriate.

## Figures and Tables

**Table 1:** Description of sample.

	Younger than 65 (*n* = 167)		65 or older (*n* = 69)		All participants (*n* = 236)	
	Mean	SD	Mean	SD	Mean	SD
Age (years)	42.81	13.46	73.90	6.01	51.90	18.42
Education (years)	13.01	2.37	13.48	2.54	13.15	2.42
TOFHLA reading^a^	45.83	4.66	45.28	7.21	45.67	5.52
General verbal ability^b^	92.38	10.40	99.22	12.24	94.38	11.38
Reading skills^c^	94.83	9.30	101.43	14.14	96.76	11.32
Digit span backwards^d^	6.72	2.45	6.19	2.05	6.57	2.35

^a^Total number of TOFHLA reading comprehension items answered correctly (range 0–50).

^b^Woodcock-Johnson Psychoeducational Battery Verbal Comprehension composite score (mean = 100; SD = 15).

^c^Woodcock-Johnson Psychoeducational Battery Passage Comprehension subtest standard score (mean = 100; SD = 15).

^d^Wechsler Adult Intelligence Scale, 3rd Ed. Digit Span Backward subtest raw score.

**Table 2:** Item difficulties, discriminations, and betas.

Item	Difficulty	SD	Discrimination	Beta^b^
A1^a^	0.99	0.09	0.60	0.03
A2	0.97	0.16	0.53	0.00
A3	0.89	0.31	0.34	0.01
A4	0.97	0.18	0.42	0.04
A5	0.98	0.13	0.35	0.03
A6	0.96	0.20	0.42	0.01
A7	0.97	0.18	0.54	0.01
A8	0.98	0.13	0.34	0.01
A9	0.94	0.24	0.26	0.01
A10	0.97	0.17	0.54	**0.05**
A11	0.98	0.13	0.42	0.02
A12	0.99	0.11	0.54	0.02
A13	0.96	0.20	0.41	0.03
A14	0.95	0.21	0.55	0.01
A15	0.97	0.17	0.38	0.01
A16	0.97	0.16	0.57	0.03
B17	0.98	0.14	0.57	0.01
B18	0.99	0.09	0.60	0.01
B19	0.83	0.37	0.27	0.03
B20	0.97	0.18	0.25	0.00
B21	0.93	0.26	0.65	0.01
B22	0.95	0.22	0.42	0.02
B23	0.96	0.19	0.47	**0.05**
B24	0.84	0.37	0.49	**0.08** ^ c^
B25	0.92	0.27	0.52	0.04
B26	0.92	0.27	0.52	0.04
B27	0.97	0.16	0.55	0.01
B28	0.97	0.16	0.58	0.02
B29	0.92	0.27	0.53	0.02
B30	0.94	0.24	0.63	0.02
B31	0.89	0.31	0.32	**0.08** ^ c^
B32	0.92	0.27	0.41	**0.05** ^ c^
B33	0.97	0.18	0.50	0.03
B34	0.49	0.50	0.19	**0.19** ^ c^
B35	0.97	0.17	0.65	0.03
B36	0.97	0.17	0.50	0.02
C37	0.97	0.18	0.67	0.02
C38	0.93	0.25	0.68	0.02
C39	0.83	0.38	0.47	**0.10** ^ c^
C40	0.84	0.37	0.52	0.03
C41	0.81	0.39	0.56	**0.07**
C42	0.92	0.27	0.56	0.02
C43	0.88	0.32	0.63	0.03
C44	0.92	0.27	0.68	0.02
C45	0.55	0.50	0.37	**0.09** ^ c^
C46	0.83	0.38	0.56	0.02
C47	0.46	0.50	0.32	**0.18** ^ c^
C48	0.86	0.35	0.57	**0.04**
C49	0.82	0.38	0.54	0.03
C50	0.88	0.32	0.36	0.04

^a^Letter prefixes before items numbers denote in which of the three test paragraphs the item is included.

^b^Items with values greater than the cutoff value of 0.0421 are in bold font, because of rounding some values of 0.04 is less than the cutoff value.

^c^Items that showed age-related DIF in a study of individuals younger and older than 50 treated for HIV infection [29].

**Table 3:** Regression models for performance on DIF items for younger and older participants.

		*B*	Std. error	*t*	*p*	*R* ^2^	Adj *R* ^2^	Significance of change with working memory
Models for participants younger than 65								
1	Intercept	−2.95	1.13	−2.61	**0.01**			
	Verbal ability	0.05	0.02	2.93	**0.004**			
	Reading	0.06	0.02	3.69	**<0.001**			
	Education	0.02	0.05	2.55	**0.01**	0.44	0.42	

2	Intercept	−2.70	1.14	−2.37	**0.2**			
	Verbal	0.04	0.02	2.71	**0.001**			
	Reading	0.06	0.02	3.43	**0.001**			
	Education	0.12	0.05	2.49	**0.01**			
	Working memory	0.06	0.05	1.36	0.18	0.44	0.43	0.18

Models for participants 65 years of age or older								
1	Intercept	0.14	1.28	0.11	0.91			
	Verbal ability	−0.01	0.02	−0.67	0.95			
	Reading	0.06	0.02	3.21	**0.002**			
	Education	0.22	0.08	2.68	**0.009**	0.55	0.52	

2	Intercept	0.30	1.23	0.24	0.81			
	Verbal	−0.003	0.02	−0.16	0.87			
	Reading	0.5	0.02	2.54	**0.01**			
	Education	0.23	0.08	2.87	**0.006**			
	Working memory	0.19	0.08	2.41	**0.02**	0.59	0.56	**0.02**
